# An immunohistochemical study of thanatophoric dysplasia type 1 after fetus autopsy examination

**DOI:** 10.1111/cga.70004

**Published:** 2025-01-08

**Authors:** Ioanna Abba Deka, Paschalis Theotokis, Maria Eleni Manthou, Angeliki Mathioudi, Evangelia Athanasiou, Soultana Meditskou

**Affiliations:** ^1^ Department of Surgical Pathology, Medical School Aristotle University of Thessaloniki Greece; ^2^ Department of Histology and Embryology, Medical School, Faculty of Health Sciences Aristotle University of Thessaloniki Greece; ^3^ 2nd Department of Neurology, University General Hospital AHEPA, Medical School Aristotle University of Thessaloniki Greece; ^4^ Histopathology Laboratory, “Microdiagnostiki” Thessaloniki Greece

**Keywords:** fetal bone marrow, fetal pathology, hematopoiesis, skeletal dysplasia, thanatophoric dysplasia type 1

## Abstract

The current case report presents the postmortem examination findings of a 17‐week‐old female fetus displaying thanatophoric dysplasia type 1 (TD‐1) due to a known fibroblast growth factor receptor 3 (FGFR3) gene mutation. Gross and X‐ray examination revealed significant abnormalities, including skeletal malformations with prominent TD‐1 femur curvature. Microscopical evaluation indicated inadequate histological growth for the gestational age, with specific organ immaturity noted in multiple hematoxylin and eosin sections from internal organs, bone from epiphyses and diaphyses levels. Immunohistochemical analysis was conducted using specific markers, such as S100, CD34, CD117, glycophorin‐C, and myeloperoxidase, to identify various hematopoietic and mesenchymal cell types. Furthermore, this report underscores the often‐overlooked aspect of fetal hematopoiesis in cases diagnosed with TD‐1, shedding light on the development of hematopoietic cells and their markers in various tissues, with a particular emphasis on the investigation of bone marrow foci in areas with incipient or no apparent ossification. Immunohistochemical identification of hematopoiesis also served as an indirect way to identify areas of incipient or abnormal ossification.

## 1 INTRODUCTION

Skeletal dysplasias, also referred to as osteochondrodysplasias, encompass a diverse range of defects impacting bone development and growth. This includes various subcategories such as osteodysplasia, chondrodysplasia, and dysostosis, with causes ranging from genetic alterations to external factors such as maternal medication or illnesses.^1^, ^2^ Some skeletal dysplasias can be fatal, constituting a limited subset among the over 450 types classified into 40 major groups based on criteria such as clinical, radiological, molecular, and phenotypic characteristics,^3^ with 92% having identified causal genes,^4^ utilizing advanced techniques like next‐generation sequencing (NGS).

Fetuses with lethal skeletal dysplasia exhibit shorter bone length, allowing early ultrasonographic diagnosis. Various imaging methods confirm diagnoses including magnetic resonance imaging (MRI), computed tomography (CT), radiography as well as molecular analysis,^5^ whereas in some cases, invasive methods are used for genetic testing, involving the acquisition of fetal tissue through procedures like NGS.^6^ The association of fibroblast growth factor receptor 3 (FGFR3) gene and autosomal dominant inheritance is particularly linked to thanatophoric dysplasia (TD).^7^ TD has two subtypes: TD‐1, with a normal‐shaped skull and curved long bones, and TD‐2, featuring a cloverleaf‐shaped skull and straight femurs, driven by over 10 FGFR3 gene mutations, responsible for over 90% of TD‐1 cases.^8^ Family history or fetal autopsy, especially in terminated pregnancies, aids in achieving a precise diagnosis.

Despite widespread awareness of delayed skeletal development in fetuses with TD,^9^ there is a scarcity of studies evaluating postmortem examination results and comparing them with term fetuses. Additionally, immunohistochemical evaluations in this context are notably absent. This study aims to provide a comprehensive overview of gross and histological findings in a 17‐week‐old female fetus with congenital defects and skeletal malformations associated with the FGFR3 gene‐linked TD‐1. An extensive histological and immunohistochemical assessment is included.

## 2 DETAILED CASE DESCRIPTION

Surgical termination of pregnancy was performed on a 17‐week‐old female fetus. The decision was based on ultrasonographic examination showing pronounced shortening of all long bones with a characteristic curvature of the femur, without evidence of heredity.

The present study was conducted in accordance with the ethical standards set forth by the Ethical Committee of the Medical School of Aristotle University of Thessaloniki. Approval for the study was granted under protocol number 6596/14.6.22.

### 2.1 Gross examination and X‐ray imaging

**FIGURE 1:**
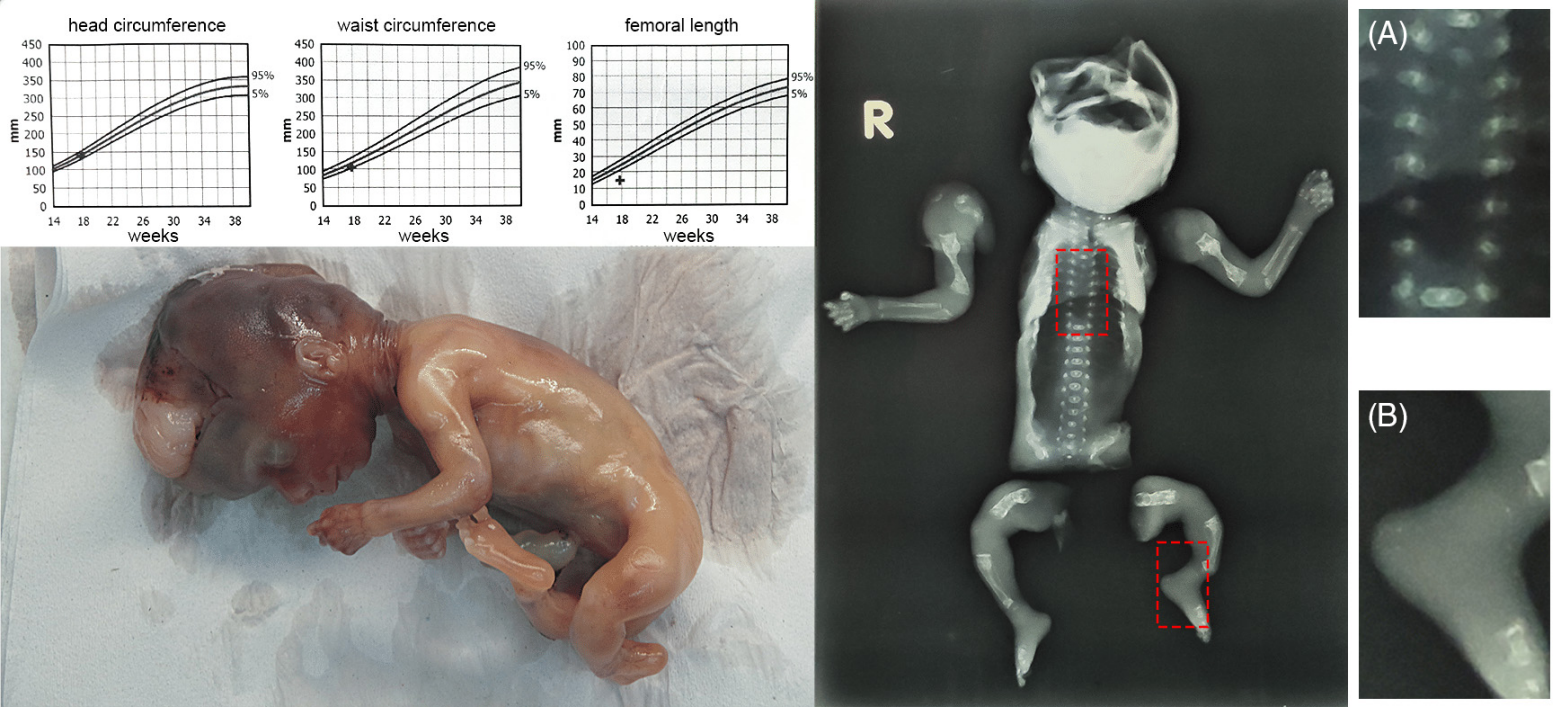
Postmortem fetal X‐rays. The X‐rays validated the gross findings, emphasizing abnormalities in the long bones of the extremities and the skull. (A) Vertebral body hypoplasia was evident at the T7, T8, and T9 levels, whereas (B) incomplete cartilage and joint growth patterns, particularly at the ankles, were noted.

During autopsy examination, congenital defects and skeletal malformations were evident, with femoral length measuring 12 mm, falling below the 5th percentile (Figure 1). Cranial bones were open with a membranous texture, displaying brain parenchyma herniation. Upper limbs were shortened with humeral bone curvature, and lower limbs showed significant shortening and arcuate deformities in femoral and tibial bones. Distal limbs lacked ligament attachment, and fibular bones were hypoplastic. Chest cavity organs, mediastinum, diaphragm, ductus arteriosus, and foramen ovale were normal. Anal atresia was absent, and abdominal pressure induced meconium flux. Other abdominal organs and hallmark structures were consistent with gestational age. Part of the umbilical cord and four placental parts were retrieved for further examination upon fetal removal.

X‐rays examination of the fetus validated the aforementioned gross findings, particularly in the long bones of the extremities and the skull (Figure 1). Vertebral body hypoplasia was additionally identified at the T7, T8, and T9 levels. Incomplete cartilage and joint growth patterns, especially at the ankles, were also noted.

### 2.2 Microscopic histochemical evaluation and genetic test

**FIGURE 2:**
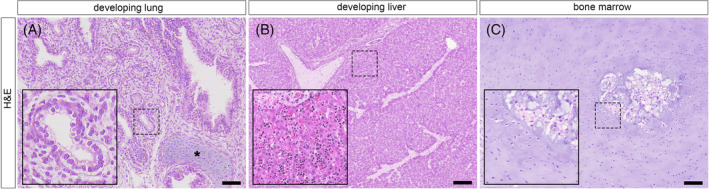
Hematoxylin–eosin histological findings for lung, liver and bone marrow development. (A) Incomplete bronchial development with pseudoglandular growth transitioning to early canalicular phase accompanied with poor vascularity. Asterisk shows bronchial cartilage. (B) Extensive liver hematopoiesis with undefined cords and underdeveloped portal triads. (C) Skeletal abnormalities showcasing disordered ossification, chondrocyte hyperplasia and incomplete column formation with reactive chondrocytes supporting hematopoiesis. Magnification insets correspond to indicative hatched boxes. Scale = 50 μm.

Placenta, membranes and the umbilical cord revealed no pathognomonic microscopic lesions. Histological sections of internal organs unveiled inadequate development for gestational age, indicating organ immaturity (Figure 2). In the lungs, pseudoglandular growth predominated, transitioning partially to an early canalicular phase, and incomplete development was noted in bronchi, with primary and a few secondary bronchi visible, covered with pseudostratified columnar ciliated epithelium, with scattered goblet cells (Figure 2). Vascularity remained poor, featuring congested large vessels. The liver showed extensive hematopoiesis, sequestered and small, with hepatocytes arranged in undefined cords and underdeveloped portal triads, while other abdominal organs appeared normal. Bone sections displayed disordered ossification, centrally located loosely structured osteoid, chondrocyte hyperplasia, and incomplete column formation, with reactive chondrocytes and foci resembling bone marrow in areas with incipient or no apparent ossification. (Figure 2).

**FIGURE 3:**
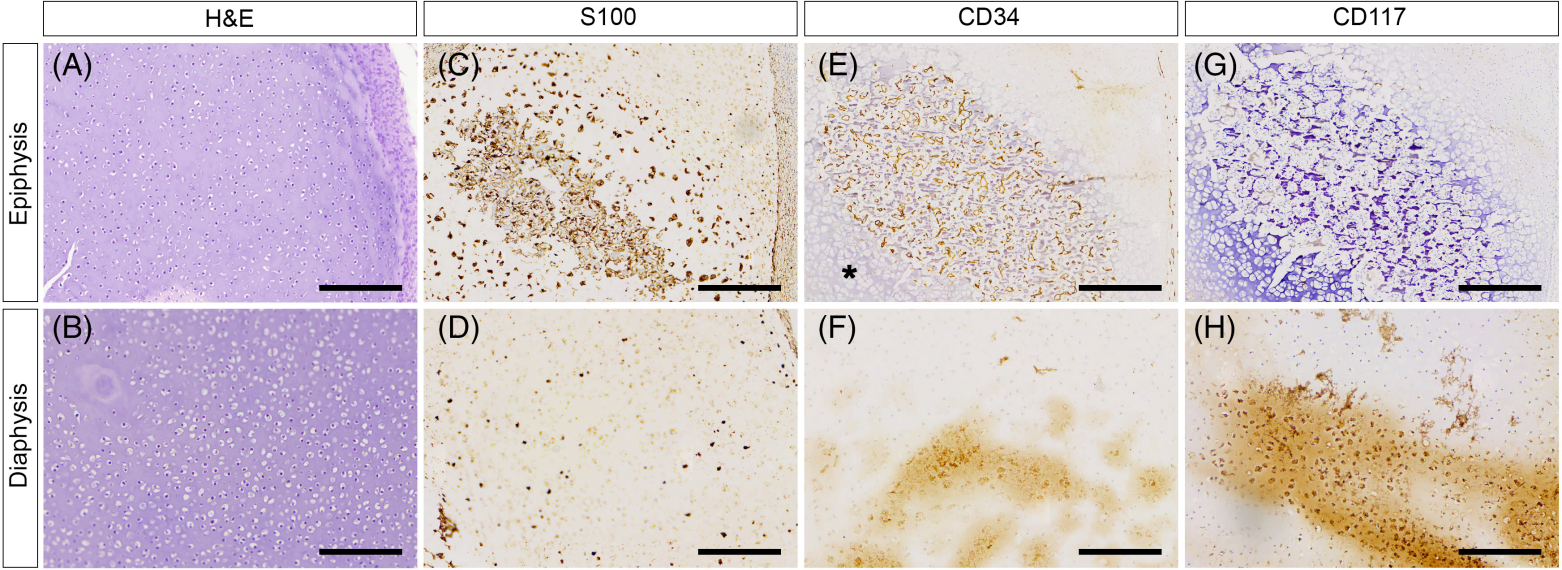
Immunohistochemical analysis with hematoxylin and eosin (H&E), S100, CD34 and CD117 for epiphyses and diaphyses of the femur bone. (A, B) H&E stain. (C, D) Positive staining for S100 protein in chondrocytes, with higher intensity and density around areas of disordered ossification, and reduced positivity in peripheral areas. (E, F) CD34 immunostaining indicated positivity around blood vessel lumens, confirming the presence of endothelial cells. In regions of disordered ossification, only a small number of CD34‐positive cells with granular cytoplasmic expression were noted. (G, H) CD117 antibody revealed weak cytoplasmic expression of myeloid hematopoietic stem cells, whereas cells maintaining a mesenchymal morphology also showed positivity for the CD117 marker. Scale = 100 μm.

**FIGURE 4:**
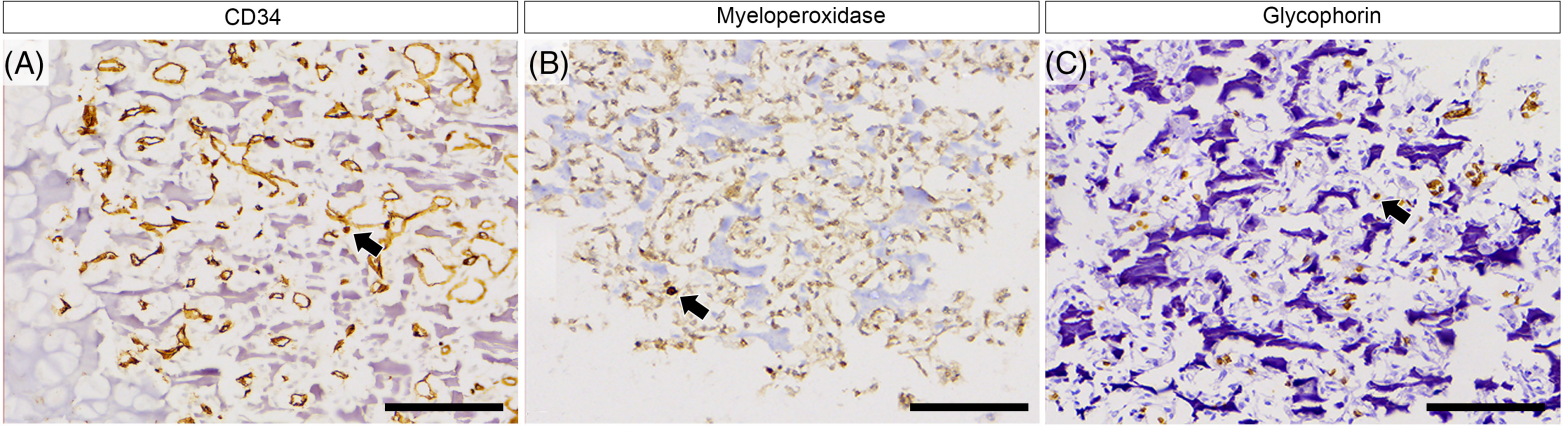
Immunohistochemical analysis with CD34, myeloperoxidase (MPO) and glycophorin‐C in the femur bone and pelvis. (A) Sparse CD34‐positive cells with granular cytoplasmic expression were observed in disordered ossification foci. (B, C) A few positively‐stained cells were observed in myeloid stem cells and nucleated erythroid cells for MPO and glycophorin‐C, respectively. Scale = 100 μm.

Immunohistochemistry using S100 antibody (Dako, IS504, polyclonal, 1:2000) positively stained chondrocytes, with a higher intensity and number around areas of disordered ossification, and less positivity in peripheral areas (Figure 3). Additionally, S100 highlighted nerve endings and plexuses at the sacral level, a well‐developed colonic myenteric plexus, and large ganglia. Anti‐CD34 immunohistochemical evaluation (Dako, M7165, monoclonal, 1:100) revealed positivity around blood vessel lumens, indicating the presence of endothelial cells. Sparse CD34 positive cells with granular cytoplasmic expression were observed in disordered ossification foci, characterized by their large, oval, and nucleated nature with scant basophilic cytoplasm and no perinuclear halo (Figure 4). Furthermore, immunohistochemical weak cytoplasmic expression with CD117 antibody (Dako, A4502, polyclonal, 1:100) was observed in cells with characteristics of myeloid hematopoietic stem cells. Cells maintaining a mesenchymal morphology, with cytoplasmic processes, also displayed positivity for the CD117 immunohistochemical marker (Figure 3).

The evaluation of immunohistochemical expression of glycophorin‐C (Dako, Re1401, monoclonal, 1:200) and myeloperoxidase (Dako, A0398, polyclonal, 1:1000) revealed positivity in nucleated erythroid and myeloid stem cells, respectively (Figure 4). As an internal positive control for glycophorin, mature non‐nucleated red blood cells stained positive, predominantly present in the lumen of adjacent vessels. Immature granulocyte cells were positioned in a paratrabecular or perivascular manner, exhibiting prominent, abundant cytoplasmic granules. Immature erythrocytes displayed an increased nuclear/cytoplasmic ratio with no present nucleolus, and no granules were observed in their cytoplasm (Figure 4).

The autopsy and pathologoanatomical results were compatible with TD. Last but not least, TD‐1 was confirmed upon genetic testing revealing that the de novo mutation is the substitution of cysteine with arginine at the protein 248 site (Arg248Cys or R248C). This mutation was identified using a targeted genetic analysis approach. Initially, DNA was extracted from fetus cells, and the specific region of the FGFR3 gene, known to harbor mutations associated with TD‐1, was amplified using polymerase chain reaction (PCR). Subsequently, the amplified DNA was subjected to Sanger sequencing, a method that allows for the precise reading of the nucleotide sequence. This sequencing process revealed the exact point mutation, where a single nucleotide change resulted in the substitution of cysteine with arginine. The identification of this specific mutation is crucial, as it confirms the diagnosis of TD‐1, providing a clear genetic basis for the clinical manifestations observed in the fetus. Since the gene responsible for thanatophoric dysplasia follows an autosomal dominant inheritance pattern,^10^ the healthy parents were not examined for the mutation, which was considered de novo.

## 3 DISCUSSION

In this case report, the postmortem examination of a 17‐week‐old female fetus with confirmed, femur‐deformed TD‐1, revealed profound developmental abnormalities across various organ systems. Femur and pelvis, which were histologically thoroughly examined, are among the specific areas of abnormal ossification which contribute to the distinct clinical, radiological and histological features associated with thanatophoric dysplasia.^5^, ^7^ TD results from a mutation in the FGFR3 gene, a fibroblast growth factor receptor located on the short arm of chromosome 4. Ordinarily, this gene exerts an inhibitory influence on endochondral ossification, slowing the formation of bone tissue. Although TD‐1 is linked to over 10 different FGFR3 gene mutations, the prevalent mutation—the one presented herein—substitutes a single aminoacid (cysteine with arginine) in the FGFR3 protein. The novelty of our findings lies in establishing a direct connection between the confirmed genetic basis of TD‐1 and the insightful immunohistochemical data, including markers such as CD34, CD117, glycophorin‐C, and myeloperoxidase (MPO) in foci of incipient or absence of ossification, between cartilage tissue.

During endochondral ossification, the proliferation of chondrocytes leads to the significant production of angiogenic factors, crucial for the formation of primary ossification centers. In our case, the identification of ossification centers or bone presence was challenging, often indirectly inferred through the observation of hemopoiesis. Notably, the primary morphological evidence for hematopoiesis in fetal bone marrow in foci without evidence of ossification was characterized by the presence of CD34 positive endothelial cells and a substantial stromal cell population, establishing a conducive microenvironment for hematopoiesis. Additionally, a nucleated CD34 positive hematopoietic stem cell was discovered in the bone marrow, a common marker for vascular and lymphohematopoietic stem and progenitor cells involved in the development of various cell lines in fetal hematopoietic tissues.^11^, ^12^

Another marker utilized to identify hematopoietic cells within the myeloid cell line was MPO. This protein is typically expressed in various tissues, including the yolk sac, liver, and bone marrow, depending on the stage of development. The presence of an enucleated cell positive for MPO in the bone marrow confirmed the existence of the myeloid cell line at that time. According to Strobl et al., MPO is detected in a significant portion, ranging from 23% to 50% of CD34 positive cells within the bone marrow.^13^ Equally important, glycophorin‐C is a protein from the glycophorin family, found on the membrane of erythrocytes. Glycophorin‐C positivity in enucleated erythroblasts was evident in the bone marrow cavity suggesting the presence of cells associated with the erythroid lineage.^14^ Erythroblasts, typically found in the bone marrow cavity, express glycophorin‐C across all maturation stages. However, current studies on glycophorin‐C expression in embryonal tissue remain exceptionally limited.

The final marker used, CD117, also known as c‐kit, revealed oval or oblong‐shaped cells probably in the bone marrow foci, displaying weak CD117 staining, indicating hematopoiesis. CD117 is a cell surface protein predominantly expressed in bone marrow stem and progenitor cells, including hematopoietic stem cells.^15^ By the 11th day of embryonic development, hematopoietic stem cells express the c‐kit marker, playing a crucial role in embryonic hematopoiesis by mobilizing and releasing progenitor cells, although the exact mechanism remains not fully understood.^16^, ^17^

In addition to the weak positivity of CD117 in myeloid hematopoietic stem cells in the bone marrow cavity, another distinct group of cells exhibited positivity at the periphery of the preformed cartilage, where they maintained a mesenchymal morphology with cytoplasmic processes, possibly originating from a common type of early mesenchymal progenitor cell.^18^, ^19^ Lastly, although He et al. documents that fetal c‐kit positive cells give rise to nearly half of all osteoblasts we postulate that the observed failure of osteoblast differentiation could be due to skeletal stem cells, which are known to cohabit with hematopoietic stem cells in the bone marrow.^20^

## 4 CONCLUSION

In conclusion, our comprehensive study of a fetus with thanatophoric dysplasia (TD‐1) highlights significant challenges in bone formation. We found that bone quality was compromised, and the maturation of progenitor mesenchymal cells into osteoblasts was impaired. Importantly, strong evidence of fetal hematopoiesis in what appeared to be hyaline cartilage suggests that abnormal osteopoiesis was occurring in the long bones, even though bone formation was not visibly apparent through histochemical staining. These findings reveal an often‐overlooked aspect of fetal hematopoiesis in TD, indicating that it can signal areas of osteopoiesis that are otherwise unrecognizable due to impaired bone formation.

## CONFLICT OF INTEREST STATEMENT

The authors declare no conflict of interest.

## Supporting information


**APPENDIX S1:** Supporting information.
