# Supplementary material for: An immunohistochemical study of thanatophoric dysplasia type 1 after fetus autopsy examination
Source: Congenit Anom (Kyoto). 2025 Jan 8;65(1):e70004. doi: 10.1111/cga.70004 (PMC11710925; doi:10.1111/cga.70004)
Supplement: Supplementary file 1 — APPENDIX S1: Supporting information. [file CGA-65-0-s001.docx]

**Materials and Methods: Immunohistochemical Staining Protocol**

Tissue specimens were fixed in 10% formaldehyde solution for 24 hours at RT, followed by routine histological processing. This process included dehydration of the tissue and embedding it in paraffin wax blocks. The embedded tissues were sectioned at a thickness of 4 μm using a microtome, and the sections were mounted on negative-charged glass slides. Deparaffinization of the slides was carried out in a heat chamber for 12 hours to facilitate optimal heat-induced epitope retrieval. Slides were then incubated in PBS for 10 min, proteins were blocked for 5 min by a blocking buffer. Specimens were then incubated with the following primary antibodies: Polyclonal Rabbit Anti-S100 (Dako; IS504, 1:2000), Monoclonal Mouse Anti-Human CD34 Class II/QBEnd-10 (Dako; M7165, 1:100), Polyclonal Rabbit Anti-Human CD117/c-kit (Dako; A4502, 1:100), Monoclonal Mouse Anti-Human Glycophorin C/clone Ret40f (Dako; Re1401, 1:200), Polyclonal Rabbit Anti-Human Myeloperoxidase (Dako; A0398, 1:1000). The staining process was automated using the DAKO Autostainer, with primary antibodies applied at various dilutions, as recommended by the manufacturer, overnight. Slides were then incubated with secondary antibodies (biotinylated goat anti-mouse IgG and goat anti-rabbit IgG) diluted 1:200 in PBS, as recommended by the manufacturer. Section staining was completed by adding DAB dye for 1 minute, washing with distilled water and then adding hematoxylin and eosin for 2 min. After staining, the slides were dehydrated by passing them through increasing concentrations of alcohol, followed by xylene. Finally, DPX mounting medium was applied, and coverslips were placed for microscopic examination.
